# Systematic literature review of integrated community case management and the private sector in Africa: Relevant experiences and potential next steps

**DOI:** 10.7189/jogh.04.020414

**Published:** 2014-12

**Authors:** Phyllis Awor, Jane Miller, Stefan Peterson

**Affiliations:** 1 School of Public Health, College of Health Sciences, Makerere University, Kampala, Uganda; 2 Centre for International Health, Global Public Health and Primary Care, University of Bergen, Norway; 3 Malaria and Child Survival Department, Population Services International, Nairobi, Kenya; 4 Global Health, Karolinska Institutet, Stockholm, Sweden; 5 International Maternal and Child Health Unit, Uppsala University, Uppsala, Sweden

## Abstract

**Background:**

Despite substantial investments made over the past 40 years in low income countries, governments cannot be viewed as the principal health care provider in many countries. Evidence on the role of the private sector in the delivery of health services is becoming increasingly available. In this study, we set out to determine the extent to which the private sector has been utilized in providing integrated care for sick children under 5 years of age with community–acquired malaria, pneumonia or diarrhoea.

**Methods:**

We reviewed the published literature for integrated community case management (iCCM) related experiences within both the public and private sector. We searched PubMed and Google/Google Scholar for all relevant literature until July 2014. The search terms used were “malaria”, “pneumonia”, “diarrhoea”, “private sector” and “community case management”.

**Results:**

A total of 383 articles referred to malaria, pneumonia or diarrhoea in the private sector. The large majority of these studies (290) were only malaria related. Most of the iCCM–related studies evaluated introduction of only malaria drugs and/or diagnostics into the private sector. Only one study evaluated the introduction of drugs and diagnostics for malaria, pneumonia and diarrhoea in the private sector. In contrast, most iCCM–related studies in the public sector directly reported on community case management of 2 or more of the illnesses.

**Conclusions:**

While the private sector is an important source of care for children in low income countries, little has been done to harness the potential of this sector in improving access to care for non–malaria–associated fever in children within the community. It would be logical for iCCM programs to expand their activities to include the private sector to achieve higher population coverage. An implementation research agenda for private sector integrated care of febrile childhood illness needs to be developed and implemented in conjunction with private sector intervention programs.

Despite substantial investments made over the past 40 years in low income countries, governments cannot be viewed as the principal health care provider in many countries [1]. Evidence on the role of the private sector in the delivery of health services is becoming more available [2,3].

Integrated community case management (iCCM) of malaria, pneumonia and diarrhoea is a public sector strategy aimed at improving timely access to treatment for sick children in resource limited settings [4]. It is now being scaled up across the African continent, largely by means of community health workers. However, in many low income countries, the first source of care for most children with fever is usually the private sector, mainly comprising of small drug shops which sell medicines as a business [5-8]. The quality of care provided at this level is also known to be low [7,9].

We set out to determine the extent to which the private sector has been utilized in providing integrated care for sick children under–5 years of age with community–acquired malaria, pneumonia or diarrhoea.

## METHODS

Where relevant, we followed the Preferred Reporting Items for Systematic Reviews and Meta–Analyses (PRISMA) statement and checklist in designing and reporting our review [10]. We reviewed the published literature for iCCM related experiences within both the public and private sector. We searched PubMed and Google/Google Scholar for all relevant literature until July 2014. The search terms used were “malaria”, “pneumonia”, “diarrhoea”, “private sector” and “community case management”. In PubMed, we used the advanced search option. We combined the search terms and searched for private sector and public sector studies, respectively, using the following search phrases: “(((malaria) OR pneumonia) OR diarrhoea) AND private sector” and “(((diarrhoea) OR pneumonia) OR malaria) AND community case management.”

In the first step, we screened the titles of all the articles retrieved from both searches. The abstracts of the titles that included malaria, pneumonia or diarrhoea in the public or private sector were then selected and read. Finally, for articles where abstracts reported results from evaluation studies that met our inclusion criteria, we read through the full text to confirm this. Wherever clarification was needed, we re–read through the full text of the relevant articles. We included all peer–reviewed studies reporting the evaluation of any intervention with drugs and or diagnostics for malaria, pneumonia or diarrhoea, or a combination of those illnesses in children within the private or public sector. We included the following types of studies: randomized controlled trials, quasi experimental studies, and studies with a pre–post design with or without a control group. We also accessed grey literature by searching websites of organizations involved in private sector work. The number and characteristics of studies in both private and public sector, reporting iCCM–related interventions either separately or in an integrated manner, are reported.

## RESULTS

**Figure 1:**
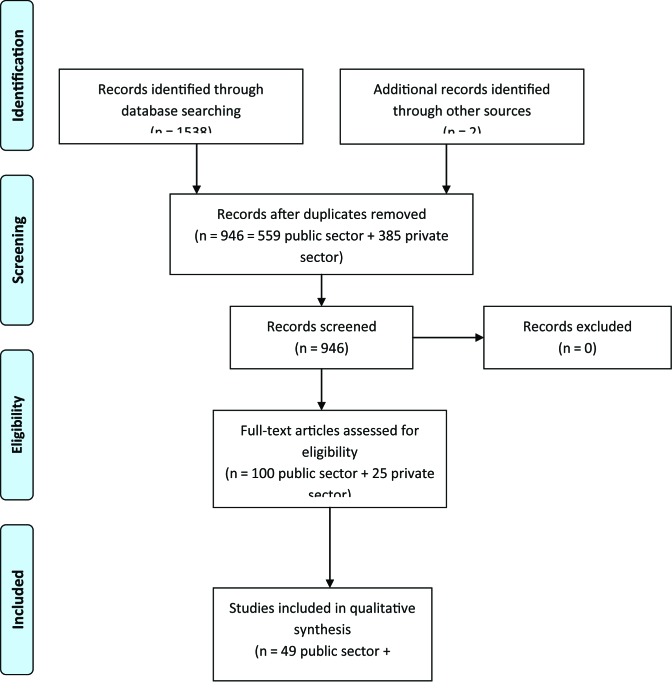
Flow diagram of literature search and screening.

A total of 944 papers were found by searching the databases. These included 385 private sector and 559 public sector papers. An additional 2 papers were included from the grey literature. After screening, 13 private sector and 49 public sector papers remained for final analysis (**Figure 1**). The final papers included were from studies conducted in 20 countries: 44 in Africa, 16 in Asia and 2 in Latin America. Most studies were conducted in rural settings.

We found 385 articles referring to malaria, pneumonia or diarrhoea in the private sector. The majority of these studies (290) were only malaria related. Thirteen studies met the inclusion criteria (**Table 1**) and most of them (86%) evaluated only introduction of malaria drugs and/or diagnostics into the private sector [11-21]. There were no articles reporting community level interventions for pneumonia treatment or diagnosis within the private sector. Only one study evaluated a diarrhoea treatment intervention, using both private and public sector [22].

**Table 1:** Studies reporting evaluation of interventions for malaria, pneumonia or diarrhoea in the private sector, by disease category

Disease(s)	Author	Title
**Malaria**	Thomson et al. 2014 [11]	Has Tanzania embraced the green leaf? Results from outlet and household surveys before and after implementation of the Affordable Medicines Facility–malaria
	Ikwuobe et al. 2013 [12]	The impact of rapid malaria diagnostic tests upon anti–malarial sales in community pharmacies in Gwagwalada, Nigeria
	Kangwana et al. 2013 [13]	The effect of an anti–malarial subsidy programme on the quality of service provision of artemisinin–based combination therapy in Kenya: a cluster–randomized, controlled trial
	Tougher et al. 2012 [14]	Effect of the Affordable Medicines Facility—malaria (AMFm) on the availability, price, and market share of quality–assured artemisinin–based combination therapies in seven countries: a before–and–after analysis of outlet survey data
	Talisuna et al. 2012 [15]	Closing the access barrier for effective anti–malarials in the private sector in rural Uganda: consortium for artemisin–based combination therapy (ACT) private sector subsidy (CAPSS) pilot study
	Yeung et al. 2011 [16]	Socially marketed rapid diagnostic tests and ACT in the private sector: ten years of experience in Cambodia
	Littrel et al. 2011 [17]	Case management of malaria fever in Cambodia: results from national anti–malarial outlet and household surveys
	Rutta et al. 2011 [18]	Increasing access to subsidized artemisinin–based combination therapy through accredited drug dispensing outlets in Tanzania
	Kangwana et al. 2011 [19]	The impact of retail–sector delivery of artemether–lumefantrine on malaria treatment of children under five in Kenya: a cluster randomized controlled trial
	Alba et al. 2010 [20]	Improvements in access to malaria treatment in Tanzania after switch to artemisinin combination therapy and the introduction of accredited drug dispensing outlets – a provider perspective
	Abuya et al. 2010 [21]	Evaluating different dimensions of programme effectiveness for private medicine retailer malaria control interventions in Kenya
**Pneumonia**		No articles
**Diarrhoea**	Larson et al. 2012 [22]	Scaling up zinc treatment of childhood diarrhoea in Bangladesh: theoretical and practical considerations guiding the SUZY Project
**Malaria, pneumonia and diarrhoea integrated care**	Awor et al. 2014 [23]	Increased access to care and appropriateness of treatment at private sector drug shops with integrated management of malaria, pneumonia and diarrhoea: a quasi–experimental study in Uganda

Moreover, there were limited iCCM–related experiences in the private sector within the published literature, where diagnosis of non–malaria fever was made and alternative treatment provided to sick children. We are aware of one study introducing diagnostics and pre–packaged drugs for malaria, pneumonia and diarrhoea into private sector drug shops [23]. Population Services International (PSI) is implementing iCCM in several countries [24] and Larsen et al. (in preparation) will report on improving quality of private sector case management of diarrhoea, pneumonia and malaria in Uganda using a Social Franchising approach.

In contrast, of 559 articles retrieved when we searched for iCCM–related experiences in the public sector, the majority were directly related to CCM of 2 or more illnesses (malaria, pneumonia and diarrhoea). Forty nine articles met the inclusion criteria of studies evaluating an intervention with drugs or diagnostics in the community, for malaria, pneumonia or diarrhoea. Only 13 (26%) of the included studies on iCCM in the public sector evaluated an intervention for malaria only (**Table 2**).

**Table 2:** Studies reporting evaluation of interventions for malaria, pneumonia or diarrhoea in the public sector, by disease category

Disease(s)	Author	Title
**Malaria**	Siekmans et al. 2013 [25]	Community case management of malaria: a pro–poor intervention in rural Kenya
	Ndiaye et al. 2013 [26]	Community case management in malaria: review and perspectives after four years of operational experience in Saraya district, south–east Senegal
	Blanas et al. 2013 [27]	Barriers to community case management of malaria in Saraya, Senegal: training, and supply–chains
	Thiam et al. 2012 [28]	Scale–up of home–based management of malaria based on rapid diagnostic tests and artemisinin–based combination therapy in a resource–poor country: results in Senegal
	Lim et al. 2012 [29]	Promoting community knowledge and action for malaria control in rural Cambodia: potential contributions of Village Malaria Workers
	Tine et al. 2011 [30]	Impact of combining intermittent preventive treatment with home management of malaria in children less than 10 years in a rural area of Senegal: a cluster randomized trial
	Chanda et al. 2011 [31]	Community case management of malaria using artemisin–based combination therapy (ACT) and rapid diagnostic tests (RDT) in two districts in Zambia: achieving high adherence to test results using community health workers
	Mubi et al. 2011 [32]	Malaria rapid testing by community health workers is effective and safe for targeting malaria treatment: randomised cross–over trial in Tanzania
	Nusungwa–Sabiiti et al. 2007 [33]	Home–based management of fever and malaria treatment practices in Uganda
	Chinbuah et al. 2006 [34]	Feasibility and acceptability of the use of artemether–lumefantrine in the home management of uncomplicated malaria in children 6–59 month–old in Ghana
	Kolaczinski et al. 2006 [35]	Adherence of community caretakers of children to pre–packaged antimalarial medicines (HOMAPAK) among internally displaced people in Gulu district, Uganda
	Kidane and Morrow 2000 [36]	Teaching mothers to provide home treatment of malaria in Tigray, Ethiopia: a randomised trial
**Pneumonia**	Kalyango et al. 2013 [37]	Integrated community case management of malaria and pneumonia Increases prompt and appropriate treatment for pneumonia symptoms in children under five years in Eastern Uganda
	Noordam et al. 2014 [38]	The use of counting beads to improve the classification of fast breathing in low–resource settings: a multi–country review
	Bari et al. 2011 [39]	Community case management of severe pneumonia with oral amoxicillin in children aged 2–59 months in Haripur district, Pakistan: a cluster randomised trial
	Soofi et al. 2012 [40]	Effectiveness of community case management of severe pneumonia with oral amoxicillin in children aged 2–59 months in Matiari district, rural Pakistan: a cluster–randomised controlled trial
	Sylla et al. 2007 [41]	Low level educated community health workers training: a strategy to improve children access to acute respiratory treatment in Senegal
	Kallander et al. 2006 [42]	Can community health workers and caretakers recognize pneumonia in children? Experiences from western Uganda
	Hadi et al. 2002 [43]	Diagnosis of pneumonia by community health volunteers: experience of BRAC, Bangladesh
	Mehnaz et al. 1997 [44]	Detection and management of pneumonia by community health workers––a community intervention study in Rehri village, Pakistan
	Bang et al. 1990 [45]	Reduction in pneumonia mortality and total childhood mortality by means of community–based intervention trial in Gadchiroli, India
	Khan et al. 1990 [46]	Acute respiratory infections in children: a case management intervention in Abbottabad District, Pakistan
	Fagbule et al. 1994 [47]	Acute respiratory infections in Nigerian children: prospective cohort study of incidence and case management
	Bang et al. 1993 [48]	Pneumonia in neonates: can it be managed in the community?
	Bang et al. 1994 [49]	Management of childhood pneumonia by traditional birth attendants. The SEARCH Team
**Diarrhoea**	Bhandari et al. 2005 [50]	A pilot test of the addition of zinc to the current case management package of diarrhea in a primary health care setting
	Sircar et al. 1991 [51]	An operational study on implementation of oral rehydration therapy in a rural community of West Bengal, India
	Benavides et al. 1994 [52]	An operational evaluation of the Community Oral Rehydration Units in Peru
	Gupta et al. 1994 [53]	Implementation of oral rehydration therapy (ORT): some problems encountered in training of health workers during an operational research programme
**Malaria and pneumonia**	Kalyango et al. 2013 [54]	High adherence to antimalarials and antibiotics under integrated community case management of illness in children less than five years in eastern Uganda
	Chinbua et al. 2012 [55]	Impact of community management of fever (using antimalarials with or without antibiotics) on childhood mortality: a cluster–randomized controlled trial in Ghana
	Mukanga et al. 2012 [56]	Integrated community case management of fever in children under five using rapid diagnostic tests and respiratory rate counting: a multi–country cluster randomized trial
	Kalyango et al. 2012 [57]	Increased use of community medicine distributors and rational use of drugs in children less than five years of age in Uganda caused by integrated community case management of fever
	Seidenberg et al. 2012 [58]	Impact of integrated community case management on health–seeking behavior in rural Zambia
	Kalyango et al. 2012 [59]	Performance of community health workers under integrated community case management of childhood illnesses in eastern Uganda
	Hamer et al. 2012 [60]	Quality and safety of integrated community case management of malaria using rapid diagnostic tests and pneumonia by community health workers
	Mukanga et al. 2011 [61]	Can lay community health workers be trained to use diagnostics to distinguish and treat malaria and pneumonia in children? Lessons from rural Uganda
	Yeboah–Antwi et al. 2010 [62]	Community case management of fever due to malaria and pneumonia in children under five in Zambia: a cluster randomized controlled trial
**Malaria and diarrhoea**	Littrell et al. 2013 [63]	Narrowing the treatment gap with equitable access: mid–term outcomes of a community case management program in Cameroon
**Diarrhoea and Pneumonia**	Puett et al. 2012 [64]	Does greater workload lead to reduced quality of preventive and curative care among community health workers in Bangladesh?
	Ghimire et al. 2010 [65]	Community–based interventions for diarrhoeal diseases and acute respiratory infections in Nepal
	Cesar et al. 2002 [66]	Changes in child health indicators in a municipality with community health workers: the case of Itapirapuă Paulista, Vale do Ribeira, Săo Paulo State, Brazil
**Malaria, pneumonia and diarrhoea**	Mugeni et al. 2014 [67]	Nationwide implementation of integrated community case management of childhood illness in Rwanda
	Yansaneh et al. 2014 [68]	Influence of community health volunteers on care seeking and treatment coverage for common childhood illnesses in the context of free health care in rural Sierra Leone
	Miller et al. 2014 [69]	Integrated community case management of childhood illness in Ethiopia: implementation strength and quality of care
	Langston et al. 2014 [70]	Plausible role for community health worker (CHW) peer support groups in increasing care–seeking in an integrated community case management project in Rwanda: a mixed methods evaluation
	Lainez et al. 2012 [71]	Insights from community case management data in six sub–Saharan African countries
	Cardemil et al. 2012 [72]	Comparison of methods for assessing quality of care for community case management of sick children: an application with community health workers in Malawi
	Chandani et. al 2012 [73]	Factors affecting availability of essential medicines among community health workers in Ethiopia, Malawi, and Rwanda: solving the last mile puzzle

## DISCUSSION

Experiences with integrated community case management of malaria, pneumonia and diarrhoea by means of public sector community health workers is increasingly reflected in the literature. Meanwhile, interventions in the private sector have so far targeted largely malaria diagnosis and management.

The community case management experience using community health workers in Africa was initiated by the Home Management of Malaria experience [36]. However, challenged by symptom overlap with other febrile illness [74] and spurred by the largely Asian success in community management of pneumonia [75], it then followed the example of integrated care in health facilities under IMCI to also become integrated in the community [4].

Meanwhile, interventions in the private sector have focused on malaria alone. This follows the historical pattern of Home Management of Malaria, and the hitherto sole malaria focus of major funders and initiatives such as the Global Fund’s Affordable Medicine Facility malaria (AMFm) [14]. From a point of quality of care to the individual sick child, as well as to make drug use more rational, it seems logical to integrate service delivery for acute febrile illness for the main causes of fever to provide alternative appropriate treatment where malaria diagnostics are negative. Here, the iCCM strategy is one vehicle. The current Global Fund application round has not quite reached integrated care of febrile illness, but it opens the door to integration with other funders towards integrated management. In March 2014, a joint statement was signed by UNICEF, Global Fund and the RMNCH Strategy Coordination Team, expressing an intention to strengthen coordination around the implementation and financing of the integration agenda, with a focus on iCCM.

However, there have been many efforts to improve quality of care in the private sector. Shah et al. reviewed the experience with different interventions, finding limited effect of the most widely used intervention model –training – suggesting to include also incentives and accountability [76]. Social franchising allows a network of independently operated health outlets to provide services and commodities to clients with oversight by a coordinating agency [77]. It provides business incentive for the health outlets and increases accountability [78], but further research is needed on the effect of social franchising on quality of care [79]. Meanwhile, the equity aspects of private sector interventions need to be clarified [80]. Also, private providers become quite context specific, requiring context–relevant interventions [8]. This implies need for further research on iCCM in private sector, and the utility of iCCM in private sector interventions, within a context of implementation research in conjunction with programs in different setting [24,81], along the lines of WHOs on–going RaCE evaluation of community health worker iCCM implementation [82].

A possible limitation of this review is that even though we tried to include all available literature/publications, some literature may not have been accessed, especially the most recent.

## CONCLUSION

While the private sector is an important source of care for children in low income countries, little has been done to harness the potential of this sector in improving access to care for non–malaria fever in children within the community. It is important for interventions and research within the private sector to provide integrated care for sick children, and not only focus on care for malaria. The iCCM strategy has the potential to act as a vehicle to improve both quality of care and make drug use more rational in the private sector, provided appropriate modification is done to reflect private sector specificities. It is also logical for iCCM programs to expand their activities to include the private sector to achieve higher population coverage. An implementation research agenda for private sector integrated care of febrile childhood illness needs to be developed and implemented in conjunction with private sector intervention programs.
